# The evaluation of pancreas β-cell autoantibodies in non-diabetic COVID-19 patients

**DOI:** 10.20945/2359-3997000000498

**Published:** 2022-06-02

**Authors:** Sanem Kayhan, Sema Hepsen, Hatice Kozan Kalkisim, Ibrahim Nahit Sendur, Fatma Aybala Altay, Ali Yalcindag

**Affiliations:** 1 University of Health Sciences Diskapi Yildirim Beyazit Training and Research Hospital Department of Internal Medicine Ankara Turkey Department of Internal Medicine, University of Health Sciences, Diskapi Yildirim Beyazit Training and Research Hospital, Ankara, Turkey; 2 University of Health Sciences Diskapi Yildirim Beyazit Training and Research Hospital Department of Endocrinology and Metabolism Ankara Turkey Department of Endocrinology and Metabolism, University of Health Sciences, Diskapi Yildirim Beyazit Training and Research Hospital, Ankara, Turkey; 3 University of Health Sciences Diskapi Yildirim Beyazit Training and Research Hospital Department of Infectious Diseases and Clinical Microbiology Ankara Turkey Department of Infectious Diseases and Clinical Microbiology, University of Health Sciences, Diskapi Yildirim Beyazit Training and Research Hospital, Ankara, Turkey; 4 University of Health Sciences Diskapi Yildirim Beyazit Training and Research Hospital Department of Clinical Biochemistry Ankara Turkey Department of Clinical Biochemistry, University of Health Sciences, Diskapi Yildirim Beyazit Training and Research Hospital, Ankara, Turkey

**Keywords:** SARS-CoV-2, COVID-19, anti-islet autoantibody, anti-GAD autoantibody, anti-insulin autoantibody

## Abstract

**Objective:**

This study aims to evaluate potential pancreas endocrine damage due to SARS-CoV-2 by measuring β-cell autoantibodies in COVID-19 patients.

**Subjects and methods:**

Between June and July 2020, 95 inpatients with a positive COVID-19 test result after polymerase-chain-reaction (PCR) and who met the inclusion criteria were enrolled in our study. Laboratory parameters that belong to glucose metabolism and β-cell autoantibodies, including anti-islet, anti-glutamic acid decarboxylase, and anti-insulin autoantibodies, were measured. β-cell autoantibodies levels of the patients were measured during COVID-19 diagnosis. Positive results were reevaluated in the 3rd month of control.

**Results:**

In the initial evaluation, 4 (4.2%) patients were positive for anti-islet autoantibody. Only one (1.1%) patient was positive for anti-glutamic acid decarboxylase autoantibody. No patient had positive results for anti-insulin autoantibody. FPG, HbA1c, and C-peptide levels were similar in patients who were split into groups regarding the initial positive or negative status of anti-islet and anti-GAD autoantibodies (p>0.05). In the 3rd month after the initial measurements, anti-islet autoantibody positivity of 2 (50%) of 4 patients and anti-glutamic acid decarboxylase positivity of 1 (100%) patient were persistent. Finally, 3 (3.1%) patients in the whole group were positive for anti-islet autoantibody in the 3rd month of control. No difference was determined between the initial and the 3rd month of parameters of glucose metabolism.

**Conclusion:**

Following an ongoing autoantibody positivity in the present study brings the mind that SARS-CoV-2 may be responsible for the diabetogenic effect. Clinicians should be aware of autoantibody-positive DM as a potential autoimmune complication in patients with SARS-CoV-2.

## INTRODUCTION

Severe acute respiratory syndrome coronavirus-2 (SARS-CoV-2) pandemic that was called Coronavirus Disease 2019 (COVID-19) by the World Health Organization (WHO) first emerged in December 2019 in Wuhan, China (1,2). The pancreatic islet is a target tissue of the SARS-CoV-2 driven by ACE2 expression (3). The entry and propagation of this virus depend on the binding of its spike glycoprotein to the ACE2 receptor present in several host organs (4).

Several studies in the field suggest that diabetes mellitus (DM) is a significant risk factor for COVID-19 and has an association with poor prognosis; the influence of COVID-19 on glycemic parameters remains unclear (5–7). Previous case reports have been presented to the literature showing the possible impact of the direct cytotoxic effect of SARS-CoV-2 on pancreatic β-cells and leading to the development of autoantibody negative insulin-dependent DM. However, the importance of ACE2 on the intra-islet paracrine mechanism is debatable, and there is no clear data available showing the association between SARS-CoV-2 infection and insulin-dependent DM (8). Additionally, the increased incidence of diabetic ketoacidosis in children and adolescents reveals a possible association between COVID-19 and newly-onset type 1 DM (9). SARS-CoV infection damages pancreatic islets and also causes subsequent acute DM (3). Moreover, it also leads to significant changes in the whole metabolism, including glucose, fat, and protein metabolism (10). Chronic inflammation affects systemic glucose homeostasis and contributes to hyperglycemia (11).

As is known, various viruses such as enteroviruses, coxsackie B virus, retroviruses, rubella, mumps, cytomegalovirus, Epstein-Barr, and varicella-zoster virus have played a role in the development of DM (12). Viral infections cause type 1 DM by triggering the production of cross-reactive antibodies due to molecular mimicry or by activating cross-reactive T cells (13). This situation has not been defined for coronavirus yet. According to a study conducted in a tertiary hospital in the United States, a significantly increased incidence of new-onset type 1 DM is observed during the COVID-19 pandemic compared to the previous years (14). Likewise, an increased type 1 DM incidence was established in a German population-based study during the COVID-19 pandemic, which may be explained by β-cell autoimmunity due to the COVID-19 virus (15). The aim of this study is to draw attention to potential pancreas endocrine injury due to SARS-CoV-2 by measuring β-cell autoantibodies levels in COVID-19 patients.

## SUBJECTS AND METHODS

### Study design

This study was designed as a prospective observational study. The Ethics Committee of our institute approved this study regarding the principles of the Declaration of Helsinki (Date: 08.06.2020/Number: 89/02). Written informed consent of all patients was obtained before inclusion.

### Patient selection

The patients diagnosed with COVID-19 between June and July 2020 were evaluated in terms of eligibility for inclusion in the study. A confirmed case of COVID-19 was defined by a positive result on a polymerase-chain-reaction (PCR) assay of a specimen collected on a nasopharyngeal swab. One hundred and five inpatients over 18 years of age who accepted the invitation were evaluated for suitability to be included in this study. The patients diagnosed with DM before and/or after hospitalization, who were started steroid or tocilizumab/anakinra treatment (drugs which can stimulate immunologic response), and pregnants were excluded. Finally, 95 patients were included in the study.

### The evaluation of demographics and laboratory parameters of the patients

Demographic data, additional diseases, and the severity of the clinical condition of the patients with a positive test result for COVID-PCR were recorded. Hemogram, kidney and liver enzymes, fibrinogen, C-reactive protein, ferritin, and D-dimer levels were evaluated. The clinical condition was categorized as asymptomatic, mild (no pneumonia or mild pneumonia), moderate/severe (dyspnea, respiratory frequency ≥ 30/min, blood oxygen saturation ≤ 93%, partial pressure of arterial oxygen to fraction of inspired oxygen ratio < 300, and/or lung infiltrates > 50% within 24/48 h), and critical (respiratory failure, septic shock, and/or multiple organ dysfunction or failure) (16).

In order to evaluate the glucose metabolism of the patients, fasting plasma glucose (FPG), HbA1c, C-peptide and anti-islet, anti-GAD, and anti-insulin autoantibodies were measured. Peripheral blood samples were collected between 8:00 and 10:00 am after at least 8-hour of overnight fasting in the first 3 days of hospitalization.

The spectrophotometric method was used to assess FPG levels (Roche Cobas 8000, Tokyo, Japan). HbA1c levels were determined through high-performance liquid chromatography (HPLC) (Bio-Rad Variant-2, Tokyo, Japan). C-peptide levels were obtained by chemiluminescent microparticle immunoassay (CMIA) method (Abbott, Architect I 2000 – Illinois, the USA), and the reference range was 0.9-7.1 ng/mL. Radioimmunoassay (Stratec-Gama Reader RIA Mass, Birkenfeld, Germany) was used for the measurement of β-cell autoantibody levels as anti-islet autoantibody, anti-insulin autoantibody, and anti-glutamic acid decarboxylase autoantibody (anti-GAD). Normal ranges of β-cell autoantibodies were as follows: >2 U/mL: positive; <1 U/mL: negative; 1-2 U/mL: borderline for anti-islet autoantibody; >2 U/mL: positive; <1 U/mL: negative; 1-2 U/mL: borderline for anti-GAD autoantibody; <8.2%: negative; >8.2%: positive for anti-insulin autoantibody. Borderline antibody results were analyzed for the second time, and the confirmed antibody statutes of the patients were presented. Antibody levels of those patients with positive or borderline antibody results and parameters related to glucose metabolism were repeated in the 3rd month after the first measurement.

### Statistical analysis

Statistical analyses were performed using SPSS software version 21 (Chicago, IL). The variables were assessed through visual (histograms, probability plots) and analytic methods (Kolmogorov-Smirnov/Shapiro-Wilk's test) to determine whether they were normally distributed or not. While the Student's t-test was performed to compare normally distributed parameters, the Mann-Whitney U test was used to compare non-normally distributed ones. Descriptive analyses were demonstrated using means and standard deviation for normally distributed variables, whereas medians and interquartile ranges (IQR) 25 and 75 percentiles were used for non-normally distributed variables. The Chi-square test or Fisher's exact test, where appropriate, was used to compare proportional data. The Kruskal-Wallis test was performed to compare non-normally distributed variables regarding the presence of autoantibodies. One-way ANOVA was used to compare normally distributed variables among the autoantibody statuses. P-values, as well as p-trend, were calculated when one-way ANOVA was used. Paired Student's t-test was used to compare the measurements at two-time points (baseline and 3 months). A p-value less than 0.05 was considered to show a statistically significant difference. When the overall significance was observed, pairwise posthoc tests were performed.

## RESULTS

Out of 105 patients meeting inclusion criteria, 6 patients previously diagnosed with type 2 DM, 2 patients using chronic steroids, and 2 patients diagnosed with DM during hospitalization were excluded. Finally, 95 patients were enrolled in the study.

The median age of the patients was 39 (IQR 25-75; 26-50) years, and 58 (61.6%) of them were female. Sixteen (16.8%) of the patients were active smokers. While 8 (8.4%) patients had a family history of type 2 DM, no patients had relatives with type 1 DM. Demographics, comorbidities, and clinical features of the participants are presented in Table 1.

**Table 1:** Demographics, clinical features, laboratory test results, and initial β-cell autoantibody levels of the patients

Patient, n	95
**Comorbidities**	
Hypertension, n (%)	14 (14.7)
Pulmonary diseases, n (%)	7 (7.4)
CAD, n (%)	1 (1.1)
**Clinical status**	
Asymptomatic, n (%)	40 (42.1)
Mild, n (%)	48 (50.5)
Moderate/severe, n (%)	7 (7.4)
**Initial laboratory test results**	
WBC	5,110 (3,880-5,930)
Neutrophils	3,029 ± 1,245
Lymphocytes	1,500 (1,100-1,910)
Platelets	219,505 ± 57,493
Creatinine, mg/dL	0.74 (0.62-0.91)
ALT, U/L	19 (14-29)
AST, U/L	19 (16-27)
LDH, U/L	180 (158-231)
CK, U/L	78 (56-152)
D-dimer, μg/ml	0.24 (0.2-0.4)
Ferritin, ng/mL	101 (38-183)
Fibrinogen, mg/dL	321.5 (275-388)
CRP, mg/L	3.38 (1-12)
FPG, mg/dL	92 (87-99)
HbA1c, %	5.4 ± 0.45
C-peptide, ng/mL	2.29 (1.75-3)
Anti-islet autoantibody level, U/mL	0.1 (0.1-0.1)
Anti-insulin autoantibody level, %	4.13 ± 0.75
Anti-GAD autoantibody level, U/mL	0.1 (0.1-0.1)

Categorical data were demonstrated with numbers and percentages (%). Normally distributed variables were presented as means (standard deviations). Non-normally distributed variables were presented as medians (interquartile ranges 25-75).

CAD: coronary artery disease; WBC: white blood cell count; ALT: alanine aminotransferase; AST: aspartate aminotransferase; LDH: lactate dehydrogenase; CK: creatinine kinase; CRP: C-reactive protein; FPG: fasting plasma glucose; HbA1c: glycated hemoglobin; GAD: glutamic acid decarboxylase.

The median FPG level of the patients was 92 (IQR 25-75; 87-99) mg/dL, and the mean HbA1c level was 5.4 ± 0.45% (Table 1). The median C-peptide level of the patients was 2.29 (IQR 25-75; 1.75-3) ng/mL (Table 1). Other laboratory test results, including hemogram parameters, kidney and liver functions, CK, D-dimer, ferritin, and fibrinogen, are demonstrated in Table 1.

In the initial evaluation of the patients, 4 (4.2%) and 12 (12.6%) patients were positive and borderline, respectively, for anti-islet autoantibody. Only one (1.1%) patient was determined to be positive for anti-GAD autoantibody, whereas 4 (4.2%) patients had borderline results for anti-GAD autoantibody. No patient had positive or borderline results for anti-insulin autoantibody. The initial β-cell autoantibody levels of the patients are presented in Table 1.

FPG and HbA1c levels were similar in the patients grouped according to the initial positive or negative status of anti-islet and anti-GAD autoantibodies (p>0.05 for each) (Table 2). Despite being within the normal limits, the initial C-peptide level of the patients with anti-islet or anti-GAD positivity was relatively lower than the negative ones. However, the difference was not statistically significant (p>0.05) (Table 2). There was no linear trend in FPG, HbA1c, and C peptide levels among subgroups of anti-islet and anti-GAD autoantibodies (p>0.05 for each). The clinical status of the patients was similar among negative and positive autoantibody groups (p>0.05 for each). No difference was determined between the initial and the 3rd month of FPG, HbA1c, C-peptide, and anti-islet autoantibody levels in 3 patients who had ongoing anti-islet autoantibody positivity. One patient with anti-GAD positivity was also positive for anti-islet autoantibody.

**Table 2:** Demographics and glucose metabolism parameters according to initial anti-islet and anti-GAD autoantibodies status

	Anti-islet Positive (N=4)	Anti- islet Borderline (N=12)	Anti- islet Negative (N=79)	P- Value	Anti-GAD Positive (N=1)	Anti-GAD Borderline (N=4)	Anti-GAD Negative (N=90)	P- Value
Age, years	35.5 (29-51.75)	42 (27.25-54.75)	38 (26-50)	0.819	32	40 (24-54.75)	39 (26-50)	0.912
Male/female, n	2/2	11/1*	24/55	**<0.001**	1/0	2/2	34/56	0.402
FPG, mg/dL (RR: 74-106 mg/dL)	88 (81-97)	92.5 (87-99)	93 (88-99)	0.600	100	95 (86-112)	92 (87-99)	0.379
HbA1c, % (RR: 4-6 %)	5 ± 0.4	5.5 ± 0.35	5.4 ± 0.46	0.162	4.9	5.7 ± 0.31	5.4 ± 0.45	0.244
C-peptide, ng/mL (RR: 0.9-4 ng/mL)	1.73 (1.38-2.42)	2.72 (1.86-3.97)	2.29 (1.7-2.94)	0.062	1.88	3.52 (2.6-4.4)	2.24 (1.73-2.93)	0.121
Anti-islet, U/mL	3.7 (3.3-4.8)	1.4 (1.2-1.9)	0.1 (0.1-0.1)	**<0.001**	3.28	0.7 (0.1-1.4)	0.1 (0.1-0.1)	**0.012**
Anti-GAD, U/mL	0.1 (0.1-9.3)	0.1 (0.1-0.1)	0.1 (0.1-0.1)	0.06	12.4	1.6 (1.3-1.9)	0.1 (0.1-0.1)	**<0.001**

Categorical data were demonstrated with numbers. Normally distributed variables were presented asmeans (standard deviations). Non-normally distributed variables were presented as medians (interquartile ranges 25-75).

* According to post-hoc analysis, male patients in anti-islet borderline group make the difference.

GAD: glutamic acid decarboxylase; FPG: fasting plasma glucose; HbA1c: glycated hemoglobin.

Meanwhile, a significant difference was observed in terms of gender among the groups considering anti-islet autoantibody status (p<0.001) (Table 2). Post-hoc analysis showed that the number of the males was significantly higher in the patients with borderline anti-islet autoantibody levels compared to the subjects with negative results (p<0.001) (Table 2).

**Figure 1:**
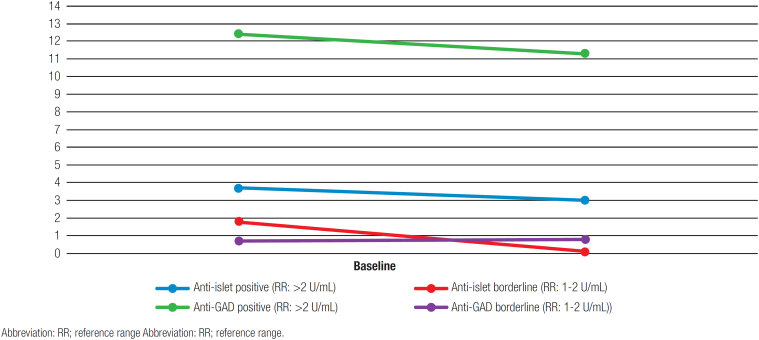
The changes in autoantibody levels at the baseline and in the 3rd-month.

In the 3rd month after the initial measurements, anti-islet autoantibody positivity of 2 (50%) of 4 patients and anti-GAD positivity of 1 (100%) patient were persistent. The other two patients with positive initial anti-islet autoantibody levels were observed in borderline. Among 12 patients having borderline results for anti-islet autoantibody at the initiation, 1 (8.3%), 7 (58.4%), and 4 (33.3%) patients were positive, negative, and borderline, respectively, in the 3rd month of control. Final 3 (3.1%) patients in the whole group were positive for anti-islet autoantibody in the 3rd month of control. While 2 (50%) of 4 patients with initial borderline results for anti-GAD remained the same, 2 (50%) of them were observed as negative. Baseline and 3rd-month autoantibody levels of the groups are demonstrated in Figure 1. Demographics and laboratory test results of the patients who had β-cell autoantibody positivity in the 3rd month are presented in Table 3.

**Table 3:** Demographics and laboratory test results of the patients with positive β-cell autoantibodies in the 3rd month

	Age (Years)	Gender	Time (Months)	FPG (mg/dL)	HbA1c (%)	C-peptide (ng/mL)	Anti-islet (U/mL)	Anti-GAD (U/mL)
Patient 1	32	Male	0	100	4.9	1.88	3.28	12.39
			3	74	5	2.32	4.9	11.3
Patient 2	43	Male	0	88	5.4	1.84	1.89	0.1
			3	95	5.6	2.57	3.95	1.18
Patient 3	56	Male	0	79	4.6	1.32	3.85	0.1
			3	81	4.8	2.13	3.9	0.1

FPG: fasting plasma glucose; HbA1c: glycated hemoglobin; GAD: glutamic acid decarboxylase.

## DISCUSSION

The present study revealed that anti-islet and anti-GAD autoantibody positivity were 4.2% and 1.1%, respectively, in COVID-19 patients in the initial evaluation. Anti-islet autoantibody positivity decreased by 3.1%, although anti-GAD autoantibody positivity remained the same in the 3rd month.

A limited number of recent studies have shown the increased incidence of type 1 DM during the pandemic (14,15). The possible reason for this increase is due to pandemic-related restriction precautions rather than the direct effect of SARS-CoV-2 on the pancreas (15). Additionally, immune-mediated and inﬂammatory responses, stress-related and steroid-induced hyperglycemia were responsible for the increase in newly diagnosed DM (17).

Viruses, one of the environmental factors, are responsible for type-1 DM and cause β-cell damage either by stimulating autoimmune attack or directly with cytotoxicity (18). Serological evidence of infection and isolation of viruses from the pancreas have been reported in a few cases recently diagnosed with DM (12,19). This situation has not been defined for coronavirus yet. However, over a decade ago, it was indicated that SARS-CoV (a cousin of SARS-CoV-2) might cause insulin-dependent DM with acute onset (3). We studied β-cell autoantibody levels in COVID-19 patients based on the idea that SARS-CoV-2 may be one of the potential environmental factors for the development of DM, although coronavirus is not on the list of viruses in type-1 DM etiopathogenesis or latent autoimmune diabetes in adults. That autoantibody positivity has remained the same (3.1%) in some patients caused worry about whether COVID-19 led to a permanent damage in pancreas islet cells when the patients detected a positive autoantibody in the inactive infection period were evaluated three months later.

SARS-CoV-2 receptor, ACE2, which plays a crucial role in the relationship between COVID-19 and hyperglycemia, is expressed in both exocrine glands and pancreas islets; therefore, pancreatic endocrine damage is expected (20). SARS-CoV-2 is likely to cause endocrine damage in the pancreas through immune-mediated mechanisms (immune-mediated cellular responses, indirect systemic inflammatory or direct cytopathic effects) due to high ACE2 concentration in the pancreas islets and also to cause insulin-dependent DM with acute onset (20,21). Apart from direct β-cell damage, alterations in self-antigens and subsequent immune-mediated destruction of β-cells could be implicated.

A limited number of cases of new-onset DM and of DM emergency were reported with COVID-19. It has led to a hypothesis that SARS-CoV-2 has a direct diabetogenic effect on pancreatic islet β-cells because of cytotoxicity (22). Additionally, much evidence was established showing the relationship between COVID-19 and other autoimmune diseases such as Kawazaki and Graves diseases (23,24). All these autoimmune complications are thought to be activated by SARS-CoV2 infection (23,25). Following ongoing autoantibody positivity in our study reminds us that SARS-CoV-2 may be responsible for the diabetogenic effect, which can reveal over an extended period by inducing autoimmunity without cytotoxic effect. The current study would provide that COVID-19 causes acute impairments in glucose metabolism and DM in the chronical process as well. For this reason, patients should be followed up for a long period.

Up till now, it has been reported that DM is closely related to increased COVID-19 risk and its poor outcomes (7,26). ACE2, which is defined as a coronavirus receptor, is expressed in pancreas β-cells, and this situation indicates that SARS-CoV-2 may cause incipient DM (27). However, the frequency of incipient DM cases and phenotypes of DM (type-1, type-2, or a new type of DM) has not been explained yet. In this study, hyperglycemia and DM were not detected in patients with autoantibody positivity at the time of diagnosis and in the 3rd month of control. Significant questions remain over whether ongoing autoantibody positivity in patients makes a clinical significance or not. β-cell autoantibody positivity may be seen in patients with preclinical type-1 DM, and it is essential for clinicians as a laboratory finding to be detected in the latent period of the disease. Based on this information, whether COVID-19 is a predisposing factor for the development of DM remains a current research topic for researchers.

Limitations: Being a single-center study, having a small sample size, and including only the Turkish population are the limitations of our study. β-cell autoantibodies, particularly anti-GAD, have been shown in the healthy population (28). That not knowing the antibody status of the patients before COVID-19 is the other limitation of our study preventing us from making a detailed comment. The lack of 3-month autoantibody levels of the patients with negative autoantibody levels initially was another limitation. The similar FPG and HbA1c results in patients with positive and negative β-cell autoantibodies could raise the question of whether these findings might be incidental as seen in normal individuals. To investigate the role of autoimmunity, evaluating these autoantibodies in patients with COVID-19 infection with new onset of DM would add more to the realistic sight of this potential association. Future studies with longer follow-up duration are needed to establish a definite relationship between autoantibody presence and DM development. However, the positivity of antibodies in these patients is an important finding for guiding future research investigating the COVID-19 and DM relationship.

In conclusion, the presence of positive β-cell autoantibody may be associated with autoimmune pancreas endocrine damage and increased DM incidence in COVID-19 patients. Clinicians should be aware of the risk of autoantibody-positive DM as a potential autoimmune complication in patients with SARS-CoV-2. Acute clinical effects of COVID-19 have been analyzed in several studies so far. The present study evaluates whether autoantibody positivity, which plays an important role in autoimmune DM development, can emerge in COVID-19. In our view, patients with COVID-19 should be followed up in terms of DM for a long period.
